# Pingyangmycin inhibits glycosaminoglycan sulphation in both cancer cells and tumour tissues

**DOI:** 10.1111/jcmm.15017

**Published:** 2020-02-18

**Authors:** Ying Lan, Xiulian Li, Yong Liu, Yanli He, Cui Hao, Hua Wang, Liying Jin, Guoqing Zhang, Shufeng Zhang, Aimin Zhou, Lijuan Zhang

**Affiliations:** ^1^ Systems Biology & Medicine Center for Complex Diseases Affiliated Hospital of Qingdao University Qingdao China; ^2^ College of Food Science and Engineering Northwest A&F University Yangling China; ^3^ College of Chemistry Tianjin Normal University Tianjin China; ^4^ Clinical Chemistry Program Department of Chemistry Cleveland State University Cleveland OH USA

**Keywords:** cancer, glycosaminoglycan, heparan sulphate, mass spectrometry, pingyangmycin

## Abstract

Pingyangmycin is a clinically used anticancer drug and induces lung fibrosis in certain cancer patients. We previously reported that the negatively charged cell surface glycosaminoglycans are involved in the cellular uptake of the positively charged pingyangmycin. However, it is unknown if pingyangmycin affects glycosaminoglycan structures. Seven cell lines and a Lewis lung carcinoma‐injected C57BL/6 mouse model were used to understand the cytotoxicity of pingyangmycin and its effect on glycosaminoglycan biosynthesis. Stable isotope labelling coupled with LC/MS method was used to quantify glycosaminoglycan disaccharide compositions from pingyangmycin‐treated and untreated cell and tumour samples. Pingyangmycin reduced both chondroitin sulphate and heparan sulphate sulphation in cancer cells and in tumours. The effect was persistent at different pingyangmycin concentrations and at different exposure times. Moreover, the cytotoxicity of pingyangmycin was decreased in the presence of soluble glycosaminoglycans, in the glycosaminoglycan‐deficient cell line CHO745, and in the presence of chlorate. A flow cytometry‐based cell surface FGF/FGFR/glycosaminoglycan binding assay also showed that pingyangmycin changed cell surface glycosaminoglycan structures. Changes in the structures of glycosaminoglycans may be related to fibrosis induced by pingyangmycin in certain cancer patients.

## 1 INTRODUCTION

Pingyangmycin, also known as bleomycin A5 (BLMA5), is a family member of bleomycin (BLM), which are a group of positively changed molecules with the same backbone structures.^1^ Pingyangmycin was discovered in 1969 at Pingyang County of Zhejiang Province of China and subsequently approved for clinical use in 1978 by the Chinese FDA.^1^, ^2^ The anticancer activities of BLMA5 are believed to rely on its abilities to produce single‐ and double‐stranded DNA breaks thus preventing DNA from replications.^3^, ^4^, ^5^ BLMA5 has largely superseded BLM as an anticancer drug in China since it is a single compound and cheaper to obtain. More importantly, BLMA5 is as potent, if not superior, in cancer treatment compared to BLM.^1^, ^6^, ^7^ However, both BLM and BLMA5 induce lung inflammation that leads to fibrosis and rapid death in certain cancer patients with unknown molecular mechanism.^4^

Glycosaminoglycans (GAGs) are linear polysaccharides comprised of glucosamine/uronic acid‐containing repeating disaccharides with different sulphation and uronic acid epimer patterns.^8^ GAGs are covalently linked to proteins during their biosynthesis. The proteins that carry GAG chains are known as proteoglycans. Two major types of GAGs are heparan sulphate (HS) consisting of GlcN‐GlcA/IdoA (glucuronic/iduronic acid) repeating disaccharide units and chondroitin sulphate (CS) consisting of GalN‐GlcA/IdoA repeating disaccharide units. HS and CS GAGs are synthesized by all animal cells. Because their repeating disaccharides can have diverse sulphation and different repeating disaccharide GlcN‐GlcA/IdoA or GalN‐GlcA/IdoA patterns due to differential expressions of GAG biosynthetic enzymes in different animal cells,^1^, ^7^, ^9^ the quantity and HS or CS disaccharide compositions are varying not only from cell to cell but also from tissues to tissues.^4^, ^10^ The biological functions of proteoglycans are largely dependent on their GAG chains.^7^, ^11^ Rapid turnover^11^, ^12^ and structure diversity generated by both biosynthesis and post biosynthesis modification allow GAGs to promote or inhibit a myriad of signalling pathways,^11^ such as fibroblast growth factor (FGF) and FGF receptor (FGFR) signalling pathway.^13^, ^14^, ^15^, ^16^, ^17^ Indeed, it has been well documented that GAGs play critical roles in physiological and pathological processes in both human and animals.^4^, ^18^, ^19^, ^20^, ^21^, ^22^, ^23^, ^24^, ^25^, ^26^, ^27^

Properly sulphated GAGs are indispensable for their biological activities. Thus, it is highly desirable to have small molecules that disrupt GAG biosynthesis in cell culture conditions or in animal models to establish GAG structure/function relationship. At present, only sodium chlorate, an inhibitor of the universal sulphate donor 3‐phospho‐adenyl‐5‐phosphosulfate (PAPS),^28^, ^29^ and D‐xylosides, which act as artificial primers of GAG biosynthesis,^29^, ^30^, ^31^ has such function. Therefore, it is important to develop proper assays to screen for reagents that disrupt GAG biosynthesis.

All BLMs are positively charged molecules and cannot get inside of cells by free diffusion.^4^ GAGs are present at the cell surface in the form of proteoglycans. Interestingly, proteoglycans are well‐established cell surface endocytosis receptors.^32^, ^33^ Therefore, we hypothesized that positively charged BLMA5 may interact with the negatively charged GAGs of proteoglycans on the cell surface for their cellular uptake. Indeed, by treating six different cell lines including a unique Chinese hamster ovary cell mutant defective in cell surface GAG biosynthesis (CHO745) with BLM A2, B2, A5 or combination of any two of them,^1^ the cytotoxicity data suggested that GAGs might be involved in the cellular uptake of all BLMs.^1^, ^4^ Since BLM has no optical properties, we have synthesized a series of small molecules with intrinsic fluorescence with or without positive charge, that is isothiouronium‐ or bromo‐modified curcumin‐pyrimidine analogs.^4^ We found that only the positively charged curcumin‐pyrimidine analogs have Golgi localization once inside of the cells.^34^ As such, we assumed that BLMA5 might interfere with GAG biosynthesis in Golgi.

Changes in CS disaccharide compositions of proteoglycans in BLM‐treated rat model have been reported.^35^ By using a novel stable isotope labelling coupled with LC/MS method, we found BLM inhibits both HS and CS sulphation in cancer cell lines.^4^ In current study, human colon cancer cell lines HCT116 and HT29, human lung cancer cell lines H1299 and A549, a Lewis lung carcinoma‐injected C57BL/6 mouse model were used to test if BLMA5 had the same effect. Wild‐type Chinese hamster ovary cell line CHOK1, cell surface GAG‐deficient CHO cell line CHO 745^36^ and 3‐O‐sulphotransferase‐1‐expressing CHOK1 cell line, CHO 3.1,^37^ were also used to test how the cell surface GAGs were related to the cytotoxicity effects of BLMA5. Furthermore, by using flow cytometry‐based cell surface FGF/FGFR/GAG binding assay plus stable isotope labelling coupled with LC/MS analysis, we demonstrated that BLMA5 inhibited glycosaminoglycan sulphation in the tumour tissues of the mouse model.

## 2 MATERIALS AND METHODS

### 2.1 Cell growth inhibition assay

The method was similar to the previously published one.^4^ Briefly, HCT116, HT29, H1299, A549 cells (Type culture collection of the Chinese academy of sciences) and Chinese hamster ovary (CHOK1, CHO 745, CHO 3.1) cells (gifts from Dr Jeffrey D. Esko, University of California, San Diego) were used to test cell growth inhibition. BLMA5 was dissolved in DMSO. Cells were seeded in 96‐well plates. After 24 hours, cells were treated with serial concentrations of the BLMA5 (10, 20, 40, 80 and 160 μmol/L) in 200 μL of complete media. After 48 hours, each well was added 20 μL, 2 mg/mL resazurin (Sigma) solution. After 16 hours of incubation at 37°C, the fluorescent signal was monitored.

### 2.2 Flow cytometry analysis of cell surface GAG/FGF/FGFR ternary complex formation

The method was similar to the previously published one.^37^ BLMA5 (10 μmol/L, 2 hours; Tianjin Tai‐he pharmaceutical), or 0.1% DMSO (Solarbio) treated A549 and HCT116 cells were collected by using phosphate‐buffered saline (137 mmol/L NaCl, 2.7 mmol/L KCl, 4.3 mmol/L Na_2_HPO_4_, 10 mmol/L MgCl_2_ and 1.4 mmol/L KH_2_PO_4_) containing 0.2% EDTA. Cells were centrifuged, and the cell pellets were placed on ice. The cells were divided into the following groups for each cell line. Control groups: 1 × 10^5^ 0.1% DMSO treated cells were mixed with: (a) 4 μg of protein A‐Alexa Fluor 488 conjugate (Thermo Fisher Scientific); (b) 1 μg of FGFR (2b or 3c)/Fc (R&D Systems) and 4 μg of protein A‐Alexa Fluor 488 conjugate; (c) 0.5 μg FGF2, FGF7, FGF8 or FGF9 (Gold biotechnology), 1 μg of FGFR (2b or 3c)/Fc and 4 μg of protein A‐Alexa Fluor 488 conjugate. Experimental groups: experimental groups: 1 × 10^5^ BLMA5 treated cells were mixed with 0.5 μg FGF (FGF2, FGF7, FGF8 or FGF9), 1 μg of FGFR (2b or 3c)/Fc and 4 μg of protein A‐Alexa Fluor 488 conjugate. Protein A binds to the Fc region of FGFR (2b or 3c)/Fc. After 15 minutes of incubation, the cells were washed once with 1 mL of phosphate‐buffered saline and re‐suspended in 0.5 mL of phosphate‐buffered saline. Flow cytometry was performed with FACScan instrument (Becton Dickinson).

### 2.3 The effects of different types of GAGs on the cytotoxicity induced by BLMA5 in cell culture

The method was similar to the previously published one.^4^ Briefly, HCT116, A549, CHO 745 cells were seeded in 96‐well plates. After 24 hours, cells were incubated with BLMA5 (15 μmol/L) in the presence or absence of varying concentrations of soluble GAG (0.15, 1.5, 15 μmol/L) for 48 hours in 200 μL of complete media. After 48 hours, each well was added 20 μL of resazurin. After 16 hours of incubation at 37°C, the fluorescent signal was monitored.

### 2.4 The effects of sodium chlorate in cell culture media on the cytotoxicity induced by BLMA5

The method was similar to the previously published one.^4^ Briefly, sulphation was inhibited by sodium chlorate treatment.^28^ HCT116, A549, CHO 745 cells were seeded in 96‐well plates. After 12 hours, cells were incubated with different concentrations (0.016‐10 mmol/L) of sodium chlorate for 12 hours in 150 μL of complete media. After 12 hours, each well was added 50 μL of complete media with or without BLM (15 μmol/L) for 48 hours. After 48 hours, each well was added 20 μL of resazurin. After 16 hours of incubation at 37°C, the fluorescent signal was monitored.

### 2.5 Purification of GAGs from control and BLMA5 treated cells

The method is the same as described previously.^4^ Briefly, control cells (0.1% DMSO treated) and BLMA5 treated cells with a final DMSO concentration of 0.1% were used for GAG preparations. GAGs were then extracted from the control and BLMA5 treated cells.

### 2.6 Establishing C57BL/6 mouse model of lung tumours

All mice were treated in accordance with the regulations approved by the Animal Ethics Committee of Ocean University of China, which were performed in accordance with National Institutes of Health guidelines.

The method is similar to that published in previous study.^38^ LLC cells (Type culture collection of the Chinese academy of sciences) were cultured in Dulbecco's modified Eagle's medium (DMEM) supplemented with 10% foetal bovine serum plus ampicillin and streptomycin routinely, and incubated in 5% CO_2_ at 37°C. The LLC cells (1 × 10^7^ cells/mL) were then injected into C57BL/6 mice to prepare enough tumour cells to establish C57BL/6 mouse model of lung tumours. Briefly, the tumour tissues from Lewis lung carcinoma mice were triturated and the tumour cell suspensions were then prepared in that the cell concentration was adjusted to 1 × 10^7^ cells/mL, and 0.2 mL cell suspensions were injected subcutaneously into the armpit of right anterior superior limbs of female C57BL/6 mice (National Institute for the Control of Biological and Pharmaceutical Products) to establish the lung tumour model, the mice were 6‐8 week old (weight 18.6 ± 0.5 g). Three days later, the mice were randomized into two groups (16 mice per group), a treatment group injected with BLMA5 in saline (1.2 mg/kg) into the hind leg muscle every 3rd day. A control group injected with the same volume of saline every 3rd day. All treatments lasted for 28 days. Mice were killed, and lung tumours were harvested and stored at −80°C until used.

### 2.7 Purification of GAGs from lung tumours

Tumours were defatted before GAG purification. For short, tumours were weighed and homogenized in cold acetone and shook at shaking table for 24 hours. The acetone was removed by aspiration after centrifugation at 3939 g for 15 minutes. Methanol and chloroform were then added to pellets while stirring to a final ratio of 1:2 (CH_3_ OH:CHCl_3_ v/v) and left overnight at room temperature followed by centrifugation at the same condition to obtain the defatted tumour tissues, which were washed twice with ethanol and dried at room temperature. The defatted tissues were suspended in 10 mL 0.25 mol/L NaCl buffer (0.25 mol/L NaCl, pH 6.0, 20 mmol/L NaAc, 0.01% Triton X‐100) before protease digestion using the identical GAG purification procedure described above.

### 2.8 Generating HS and CS disaccharide by enzymatic digestion

The method is the same as described previously.^4^ Briefly, GAGs were divided into two parts, one for HS disaccharide analysis and the other for CS disaccharide analysis. For HS digestions, GAGs were incubated with 0.33 mU each of heparin lyase I, II and III. For CS digestions, GAGs were incubated in digestion solution containing chondroitinase ABC. Digestion was monitored by using a Spectra MAX M2 plate reading spectrophotometer.

### 2.9 PMP labelling and LC/MS analysis

The method is the same as described previously.^4^ Briefly, PMP (1‐phenyl‐3‐methyl‐5‐pyrazolone) or D5PMP (2,3,4,5,6‐deuterated 1‐phenyl‐3‐methyl‐5‐pyrazolone) was added into the enzyme‐digested samples, the PMP‐ and D5PMP‐labelled products from the same amount of cellular proteins were mixed and ready for liquid chromatography coupled‐mass spectrometry (LC/MS) analysis.

## 3 RESULTS

### 3.1 BLMA5 had GAG‐dependent cytotoxicity

**Figure 1:**
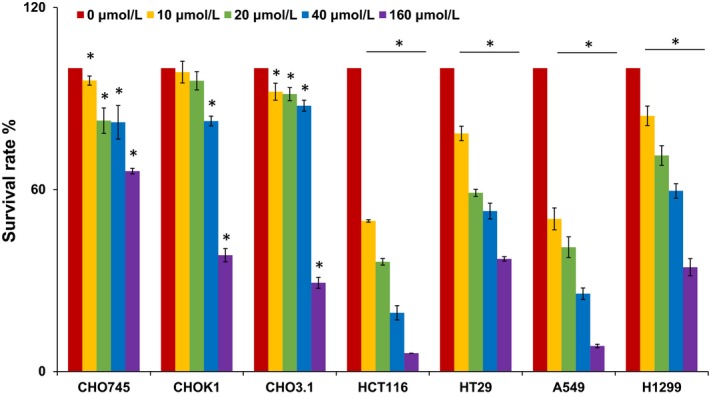
Growth inhibitory effect of BLMA5 on three CHO cell lines and four different cancer cell lines. Two human lung cancer cell lines A549 and H1299, two human colon cancer cell lines HCT116 and HT29, and three Chinese hamster ovary cell lines (CHO745, CHOK1 and CHO3.1) were used to measure the percentage of viable cells after 48 h exposure to 0‐160 μmol/L BLMA5. The experiment was repeated three times with similar results. The untreated cells (control) were assigned values of 100, and the results were presented as mean ± SD (n = 3). Significance: *P < .05 vs 0 μmol/L group

In current study, the cytotoxicity of BLMA5 on HCT116, A549, H1299, HT29, CHO K1 (wild type), CHO 745 (cell surface GAG‐deficient) and CHO 3.1 (HS 3‐O‐sulphotransferase‐1 over‐expressing) cells was analysed. The cytotoxicity at different concentrations of BLMA5 in the seven cell lines and the corresponding IC_50_ values were shown in Figure 1 and Table 1. The toxicity of BLMA5 to HCT116 was the strongest among the other cancer cell lines, which indicated that hCT2 might not be involved in the BLMA5 cellular uptake in all 7 cell lines tested since HCT116 cells do not express hCT2. The toxicity of 160 mmol/L BLMA5 to CHO K1 and CHO 3.1 cells containing GAG on the cell surface was higher than that of CHO 745 lacking GAG on the cell surface, indicating that GAG may be involved in the endocytosis of BLMA5 by cells.

**Table 1:** IC_50_ (μmol/L) values of BLMA5 in different cell lines

Cell lines	CHO 745	CHO K1	CHO 3.1	HCT116	HT29	A549	H1299
IC_50_ of BLMA5	327.7 ± 4.2	126.4 ± 2.7	122.8 ± 2.6	9.9 ± 1.6	55.4 ± 1.2	11.6 ± 1.5	71.7 ± 4

Note

Values are shown as the mean ± SD (n = 3).

### 3.2 BLMA5 treatment changed FGF/FGFR/GAG binding on the cell surfaces of A549 and HCT116 cells detected by flow cytometry

We have previously developed a flow cytometry‐based cell surface binding assay that allows to measure the interaction between cell surface GAGs with fluorescence‐labelling GAG binding proteins, such as antithrombin (Qiu et al, 2017),^37^ FGF, FGFR or combined FGF/FGFRs (Qiu et al, 2017).^39^ We reasoned that this assay would allow us to test if BLMA5 could change the cell surface GAG structures. Fibroblast growth factors (FGFs) comprise a structurally related family of 22 molecules, and bind four high affinities, ligand‐dependent FGF receptor tyrosine kinase molecules (FGFR1‐4).^40^ FGF signalling activity depends on the affinity among the FGF, FGFR and specific GAG structures.^41^

**Figure 2:**
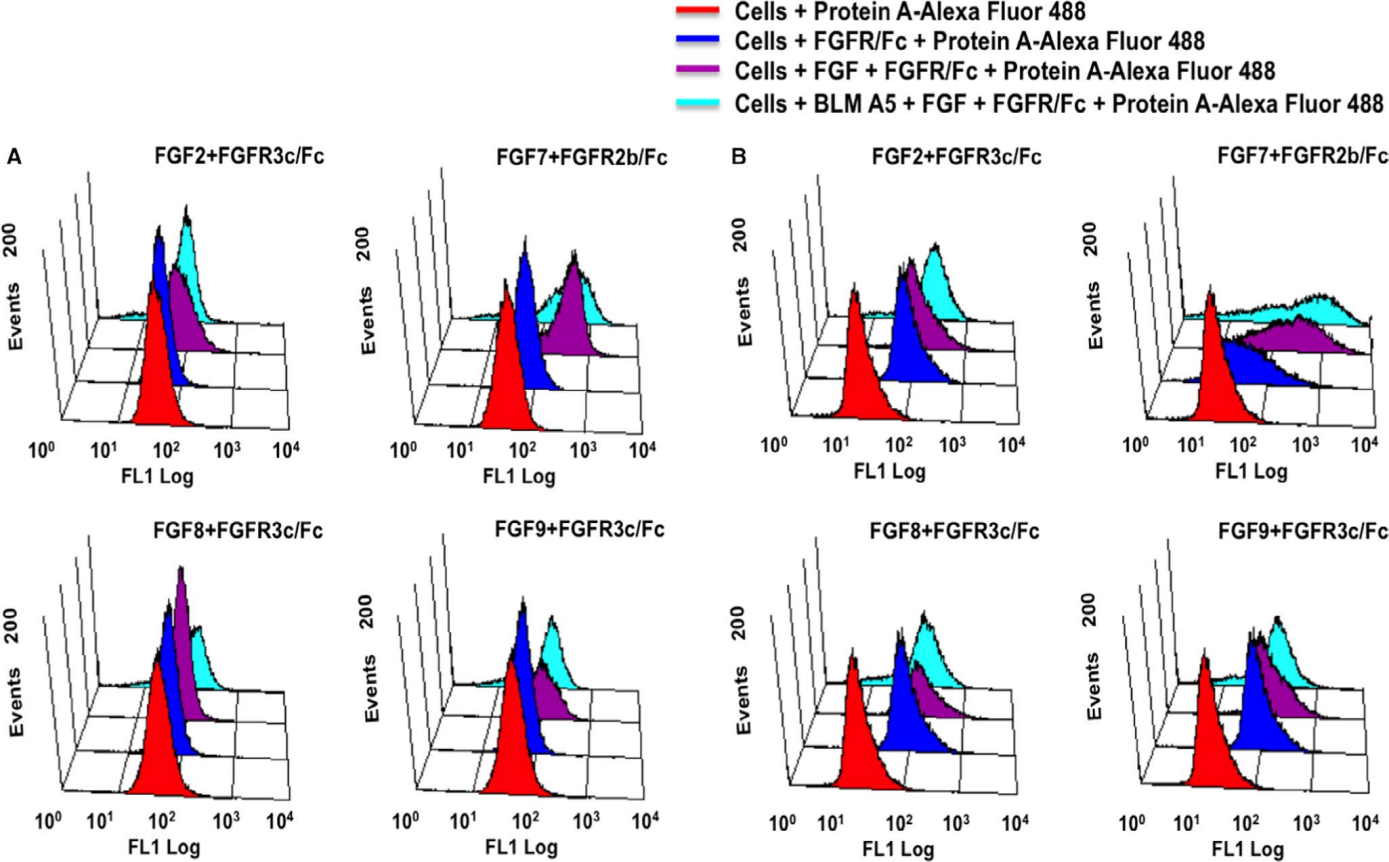
FGF/FGFR/GAG ternary complex formation on the cell surface of BLMA5 treated (10 μmol/L, 2 h) cancer cells. A, A549 cells, B, HCT116 cells

We performed the flow cytometry assay of FGF/FGFR/GAG ternary complex formation (See details in Section 2). In brief, both A549 and HCT116 cells were treated with 10 μmol/L BLMA5 for 2 hours before the assay. The protein A‐Alexa Fluor 488 was added to control cells to set up the background cell fluorescence level (Figure 2, red); the FGFR binding to control cell surface GAGs was accomplished by adding FGFR/Fc fusion protein to cells followed by adding protein A‐Alexa Fluor 488 that bond to Fc domain of the FGFR (Figure 2, blue); the FGF/FGFR/GAG ternary complex formation in control cells was conducted by adding FGF plus FGFR/Fc fusion protein to cells followed by adding protein A‐Alexa Fluor 488 (Figure 2, purple); finally, the FGF/FGFR/GAG ternary complex formation in BLMA5‐treated cells was conducted by adding FGF plus FGFR/Fc fusion protein to cells followed by adding protein A‐Alexa Fluor 488 (Figure 2, turquoise). As shown in Figure 2, both cell lines had low background fluorescence (red peaks). Interestingly, FGFR3c did not bind to cell surface GAGs of A549 (the red and blue peaks were overlapped) but FGFR3c did bind to cell surface GAGs of HCT116 (the apparent right shift of the blue peaks), which indicated that the cell surface GAGs of A549 were different from that of HCT116. In contrast, FGFR2b binds to GAGs of both A549 and HCT116 on the cell surfaces (Figure 2A,B FGFR2b, the apparent right shift of the blue peaks). Most importantly, after BLMA5‐treatment, the fluorescent intensities and profiles of all FGF/FGFR/GAG ternary complex (BLMA5 treated, turquoise peaks) were different from of that control (purple peaks) in both A549 (Figure 1A) and HCT116 (Figure 1B) cells. As is known to all, FGF7 not only binds to HS^42^ but also binds to CS‐B or dermatan sulphate,^43^ the FGF7/FGFR2b/GAG fluorescent peaks (turquoise) in both BLMA5‐treated A549 and HCT116 cells were not the same compared to that of control peaks (purple) indicated that BLMA5 treatment changed both HS and CS structures in the two cancer cell lines. Therefore, BLMA5 treatment potentially perturbed GAG biosynthesis in both cancer cell lines.

### 3.3 Cell surface GAGs were responsible for getting BLMA5 inside cells to exert its cytotoxicity

**Figure 3:**
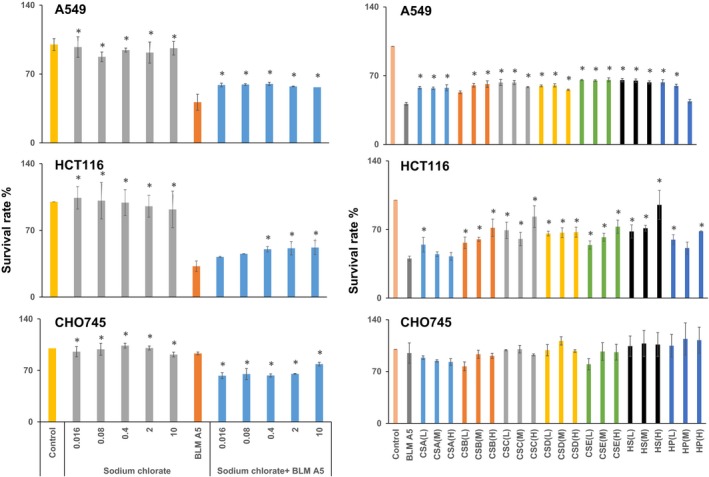
Cell surface GAGs were responsible for getting BLMA5 inside cells to exert its cytotoxicity. (a) Sulphation inhibition by sodium chlorate reduced cytotoxicity effects of BLMA5 on cancer cell lines. Human lung cancer cell line A549 and human colon cancer cell line HCT116, and Chinese hamster ovary cell line CHO745 were used to measure the percentage of viable cells after exposure to varying concentrations of sodium chlorate (0.016‐10 mmol/L) with or without BLMA5 (15 μmol/L). The experiment was repeated three times with similar results. The untreated cells (control) were assigned values of 100, and the results were presented as mean ± SD (n = 3). (b) Soluble GAG reduced cytotoxicity of BLMA5 in both HCT116 and A549 cells. Human lung cancer cell line A549 and human colon cancer cell line HCT116, and Chinese hamster ovary cell line CHO745 were used to measure the percentage of viable cells after 48 h exposure to varying concentrations of soluble GAG (0.15, 1.5, 15 μmol/L) in the presence of BLM (15 μmol/L) or BLM treatment alone (15 μmol/L). The GAGs used in the experiment include CS‐A, CS‐B, CS‐C, CS‐D, CS‐E, HS and heparin as described earlier.^44^ Since different GAGs have different molecular weight distributions and BLMA5 is a small molecule comparable to the size of a repeating disaccharide unit in the GAGs, the disaccharide concentration for each GAG was calculated based on the knowledge of its average mass of the repeating disaccharide unit. The experiment was repeated three times with similar results. The untreated cells (control) were assigned values of 100, and the results were presented as mean ± SD (n = 3). Significance: *P < .05 vs BLMA5 group

Sodium chlorate is the reversible sulphation inhibitor,^44^ and it was used to treat HCT116, A549 and CHO 745 cells to expound whether GAG sulphation was involved in the cytotoxicity of BLMA5 through endocytosis (Figure 3A‐C). Different concentrations of sodium chlorate alone (from 0.016 to 10 mmol/L) had no cytotoxicity as shown in Figure 3A‐C. However, the cytotoxicity of BLMA5 towards HCT116 and A549 cells was significantly reduced after adding different concentrations of sodium chlorate. On the contrary, at the same concentration, the cytotoxicity of BLMA5 to CHO 745 cells lacking GAG on the cell surface was significantly increased. Thus, sulphation of GAG contributed to the uptake and cytotoxicity of BLMA5 for both A549 and HCT116 cells.

To further confirm the involvement of cell surface GAG, different types of GAGs were added to cell culture medium to compete with GAGs on the cell surface for binding to BLMA5. As shown in Figure 3D‐F, the cytotoxicity of BLMA5 to HCT116 cells was negatively correlated with the concentrations of CS‐B, CS‐E and HS. Interestingly, the cytotoxicity of BLMA5 to HCT116 and A549 cells was reduced by all the GAGs at low, medium and high concentrations. On the contrary, low concentrations of CS‐B and CS‐E and high concentration of CS‐A increased cytotoxicity of BLMA5 in GAG‐deficient CHO 745 cells.

### 3.4 Changes of CS and HS disaccharides in A549 and HCT116 cells

The data in Figures 2 and 3 indicated BLMA5 treatment changed GAG structures of A549 and HCT116 cells. However, detailed GAG structure characterization is required to demonstrate such changes. In GAG research field, CS and HS disaccharide compositional analysis is used for such purpose. To facilitate quantitative comparison of GAG disaccharide compositions from different cell types, we have previously tagged the reducing end of enzyme‐digested disaccharides with aniline‐containing normal and stable isotopes.^4^, ^45^ Because different isotope tags have no effect on LC retention times but are discriminated by MS analysis, differentially isotope‐tagged disaccharides can be compared simultaneously by LC/MS.^4^, ^45^, ^46^

To test how BLMA5 changed biosynthesis and metabolism of GAGs, A549 and HCT116 cells were treated with BLMA5 either at high concentration (80 μmol/L) for a short period of time (4 hours; named HCST) and low concentration (10 μmol/L) for a long period of time (48 hours; named LCLT) for monitoring the changes of GAGs biosynthesis and metabolism, respectively (Table 1 and Figure 1). The non BLMA5‐treated cells (Control) were cultured at same time. The final DMSO concentrations in both BLMA5 and BLMA5‐free treated cells were 0.1% (See details in Section 2).

**Figure 4:**
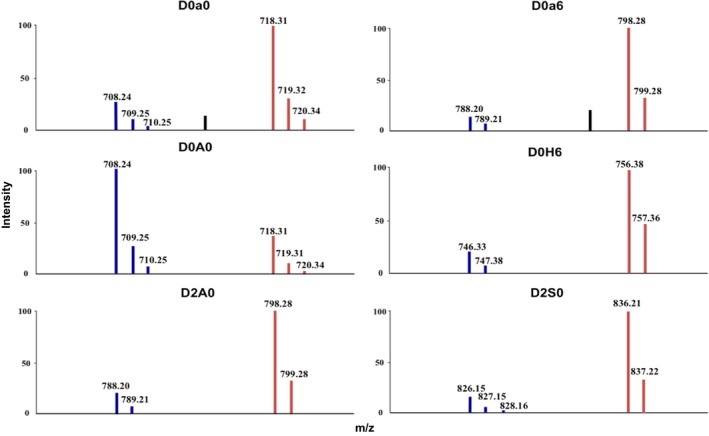
MS data of six GAG disaccharides from the PMP and D5PMP‐labelled samples from A549 cells. HS and CS disaccharides from enzymatically digested GAGs isolated from BLMA5‐treated or control A549 cells were tagged with PMP (blue, BLMA5‐treated, 80 μmol/L 4 h) or D5PMP (red, the control, 0 μmol/L 4 h), respectively. The two independently labelled samples were proportionally admixed to ensure that each sample was from the same amount of cell‐proteins. The co‐injected mixture was then subjected to LC/MS analysis. Since each disaccharide can be tagged with two molecules of PMP or D5PMP, each co‐eluted pair of PMP‐ and D5PMP‐labelled disaccharide should have a molecular weight difference of 10 in theory, which was exactly observed in the MS data shown above. All disaccharides were further identified by directly comparing both LC elution positions and *m*/*z* data with that of PMP‐labelled, commercially available disaccharide standards (See details in Section 2)

The extracted ion current of six pairs of CS and HS disaccharides from A549 cells of HCST group (PMP‐labelled, blue) and control group (D5PMP‐labelled, red) is shown in Figure 4. The ion current intensities of CS disaccharides D0a0 D0a6 decreased in the BLMA5‐treated group compared with the control group based on the same protein loading. On the contrary, the non‐sulphated HS disaccharide D0A0 was higher in the BLMA5‐treated cells whereas the monosulphated disaccharide D2A0 and D0H6, and disulphated HS disaccharides D2S0 were lower in the BLMA5‐treated cells than that in control cells. The co‐eluted pair of the stable isotope labelled disaccharide should have a 10 mass unit difference due to each disaccharide can be tagged with two molecules of PMP or D5PMP, and it was exactly observed in the MS data (Figure 4). We then summarized all the molecular mass over charge (*m*/*z*) for the stable isotope labelled GAGs disaccharides of BLMA5‐treated or non‐BLMA5‐treated cancer cells in Table 2.

**Table 2:** Molecular masses for GAG disaccharides detected in cancer cells

Disaccharide code	Structures	Observed *m*/*z* (*z* = 1)	
		PMP‐labelled	D5PMP‐labelled
CS disaccharides			
D0a0	ΔUA‐GalNAc	708	718
D0a6	ΔUA‐GalNAc6S	788	798
D0a4	ΔUA‐GalNAc4S	788	798
D2a0	ΔUA2S‐GalNAc	788	798
D0a10	ΔUA‐GalNAc4S6S	868	878
HS disaccharides			
D0A0	ΔUA‐GlcNAc	708	718
D2A0	ΔUA2S‐GlcNAc	788	798
D0A6	ΔUA‐GlcNAc6S	788	798
D0H6	ΔUA‐GlcN6S	746	756
D0S0	ΔUA‐GlcNS	746	756
D2H0	ΔUA2S‐GlcN	746	756
D0S6	ΔUA‐GlcNS6S	826	836
D2S0	ΔUA2S‐GlcNS	826	836
D2S6	ΔUA2S‐GlcNS6S	906	916

Note

The disaccharide structure code (DSC),^4^, ^56^ ΔU = 4,5‐unsaturated uronic acid.

### 3.5 The CS and HS disaccharide compositions from cancer cells

**Figure 5:**
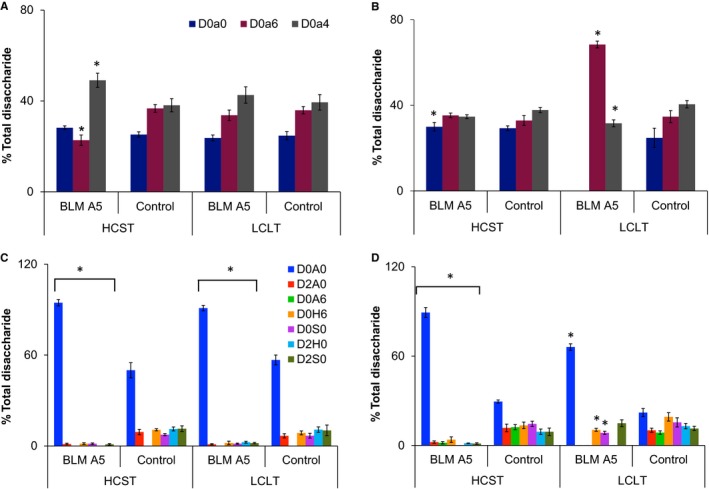
Disaccharide compositions of HS and CS from BLM A5‐treated and untreated A549 and HCT116 cells. Disaccharides from enzymatically digested HS and CS isolated from BLM A5‐treated and untreated HCT116 and A549 cells were tagged with PMP or D5PMP, respectively. The eight sets of PMP‐ or D5PMP‐labelled samples were proportionally admixed to ensure equal cell‐protein loading into four samples: A, CS disaccharide compositions of A549 cells with or without BLM A5 treatment; B, CS disaccharide compositions of HCT116 cells with or without BLM A5 treatment; C, HS disaccharide compositions of A549 cells with or without BLM A5 treatment; D, HS disaccharide compositions of HCT116 cells with or without BLM A5 treatment. The disaccharide compositions were calculated based on the MS data (mean ± SD from three independent measurements, Significance: *P < .05 vs control group). BLM A5 treatment conditions: HCST (80 μmol/L 4 h) and LCLT (10 μmol/L 48 h)

In this study, each disaccharide was separated by using a solvent gradient (See details in Section 2). The ion current intensities of the stable isotope labelled disaccharides were increased with the increase of acetonitrile concentrations during the LC run. Therefore, we used commercial disaccharides to obtain the response factor for each disaccharide using the same method we reported previously.^4^, ^11^ Then, the proportion of each GAGs disaccharide was calculated based on MS data. The results were shown in Figure 5A‐D.

First of all, CS disaccharide compositions were similar in both A549 and HCT116 control cells; however, the HS disaccharide compositions were different as the D0A6 was present in HCT116 cells but was undetectable in A549 cells (Figure 5A‐D). Secondly, the percentage of CS D0a4 disaccharide was increased in the HCST BLMA5‐treated A549 cells, whereas D0a4 disaccharide was disappeared in the LCLT BLMA5‐treated HCT116 cells. Thirdly, HS disaccharide compositions in either HCST or LCLT BLMA5 treatment conditions were totally different from that of the control A549 and HCT116 cells, that is the proportion of non‐sulphated HS disaccharides (D0A0) were increased significantly, but the proportion of all sulphated HS disaccharides were decreased, which indicated that BLMA5 suppressed HS sulphation during GAG biosynthesis. In contrast, BLMA5 treatment changed CS disaccharide compositions.

### 3.6 BLMA5 treatment reduced both HS and CS sulphation in lung tumours of LLC‐injected C57BL/7 mice

**Figure 6:**
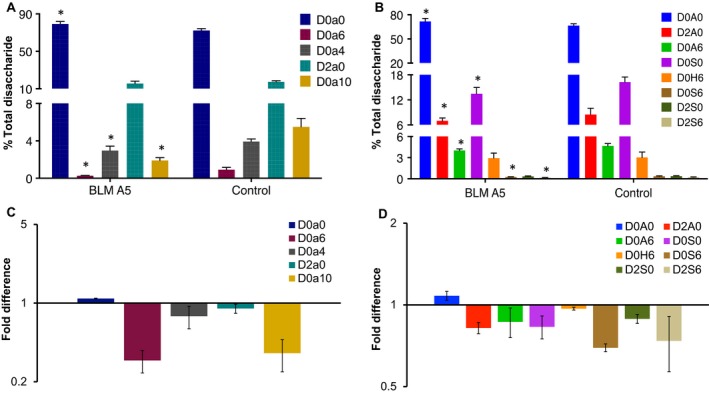
BLM A5 treatment reduced both HS and CS sulphation in lung tumours of LLC‐injected C57BL/7 mice. Disaccharides from enzymatically digested HS and CS isolated from lung tumours of BLM A5‐treated or saline‐treated LLC‐injected C57BL/6 mice were tagged with PMP or D5PMP, respectively. Samples were proportionally admixed to ensure they were from equal weight of lung tumours (BLM A5 treated groups: n = 16; control group: n = 16). A, CS disaccharide compositions of lung tumours from saline‐treated or BLM A5‐treated C57BL/6 mice; B, HS disaccharide compositions of tumours from saline‐treated or BLM A5‐treated C57BL/6 mice. C, The changes in CS disaccharides in saline‐treated vs BLM A5‐treated C57BL/6 mice; D, The changes in HS disaccharides in saline‐treated vs BLM A5‐treated C57BL/6 mice. Data are expressed as mean ± SD from three independent measurements. *P < .05 vs control group, statistical significance was determined by *t* test

The LC/MS analysis revealed that BLMA5 not only suppressed HS sulphation but also changed the quantity and sulphation pattern of CS in both A549 and HCT116 cancer cells. The next question we asked was if BLMA5 also changed HS and CS biosynthesis in lung tumours in a well‐established C57BL/6 mouse model.^38^ To this end, C57BL/6 mouse model of lung tumours was established.^38^ Three days later, the mice were divided randomly into two groups (16 mice per group), each mouse in the treatment group was injected with BLMA5 dissolved in saline and each mouse in the control group was injected with the same volume of saline (See details in Section 2). All mice were killed at 28th day, and lung tumours were harvested and weighed. As expected, BLMA5 treatment inhibited tumour growth and resulted in fewer tumours with smaller size (0.22 ± 0.16 g) compared to that of saline‐injected control mice (0.66 ± 0.46 g). Most importantly, the sulphated disaccharides in both HS and CS were decreased in the BLMA5‐treated group compared to that of saline‐treated control group. The CS disaccharide compositions of BLMA5‐treated and saline‐treated mice were shown in Figure 6A, and the HS disaccharide compositions of BLMA5‐treated and saline‐treated mice were shown in Figure 6B, respectively. The fold quantity differences in each CS (Figure 6C) and HS (Figure 6D) disaccharides from lung tumours between BLM‐treated and saline‐treated mice were calculated based on the data presented in Figure 6A,B, that is the PMP‐labelled disaccharide% from lung tumours of BLMA5‐treated C57BL/6 mice was divided by the D5PMP‐labelled disaccharide% from lung tumours of saline‐treated C57BL/6 mice. The CS D0a6 and D0a10 disaccharides (Figure 6C) were significantly decreased in lung tumours of BLMA5‐treated mice. Overall sulphation of HS disaccharides (Figure 6D) was also decreased in BLMA5‐treated mice. Thus, the data in Figure 6A‐D revealed that BLMA5 treatment reduced both CS and HS sulphation in the lung tumours of C57BL/6 mouse model significantly.

## 4 DISCUSSION

**Figure 7:**
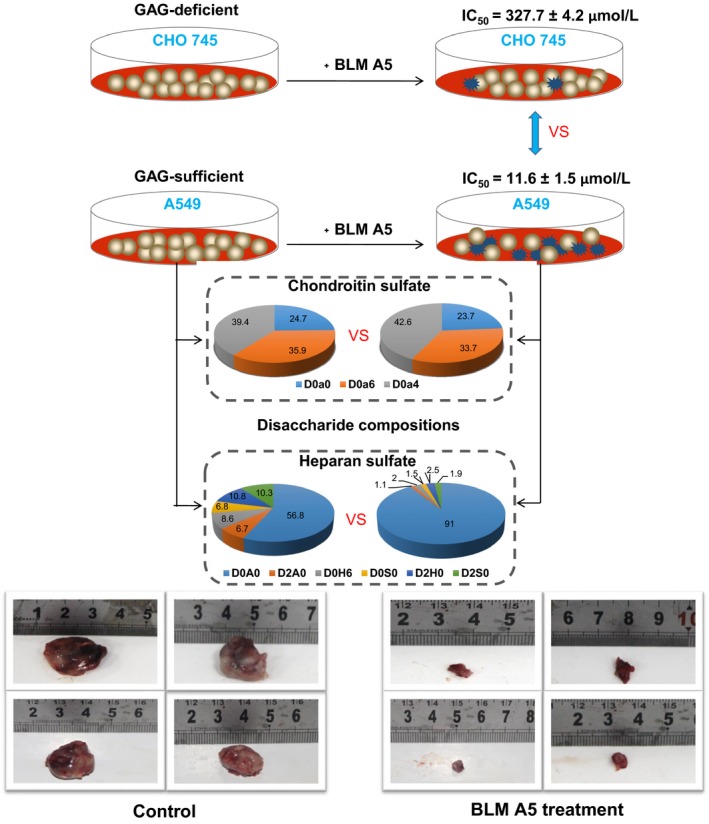
Summary of the major discoveries. D0a0, D0a6, D0a4, D0A0, D2A0, D0H6, D0S0, D2H0, D0S6 and D2S0 represent ΔUA‐GalNAc, ΔUA‐GalNAc6S, ΔUA‐GalNAc4S, ΔUA‐GlcNAc, ΔUA2S‐GlcNAc, ΔUA‐GlcN6S, ΔUA‐GlcNS, ΔUA2S‐GlcN, ΔUA‐GlcNS6S and ΔUA2S‐GlcNS, respectively

We summarized our overall results in the Figure 7 in that the level of cell surface GAG expression was correlated with the cytotoxicity of BLMA5 in CHO745 and A549 cells; both chlorate and soluble GAG‐treatment reduced the cytotoxicity of BLMA5 in A549 and HCT116 cells; HS was significantly undersulphated, both the quantity and disaccharide compositions of CS was also changed in BLMA5‐treated A549 cells; BLMA5 treatment of C57BL/6 mice resulted in smaller size of lung tumours with reduced HS and CS sulphation. BLMA5 caused undersulphation of HS both biosynthetically and metabolically as evidenced by the results obtained in two different cell culture conditions (Table 2, Figure 5A‐D). BLMA5 also changed the quantity and disaccharide compositions of CS in both HCT116 and A549 cells based on the LC/MS analysis. The effect of BLMA5 on HS and CS disaccharide compositions was similar at high and lower concentrations and at different exposure times, suggesting a strong causal effect of BLMA5. Most importantly, BLMA5 treatment not only inhibited lung tumour growth but also reduced both CS and HS sulphation in the lung tumours of LLC‐injected C57BL/6 mouse model significantly.

In Figure 5B, BLMA5 treatment led to a dramatic increase in CS D0a6 in HCT116 cells; however, D0a6 in BLMA5 treated LLC cells (Figure 6A) was significantly reduced, which raised the question how could BLMA5 have opposite effects in these two cell lines. Based on current understanding of GAG biosynthesis, different cell lines have different GAG composition and structures due to the expression of different repertoires of enzymes responsible for GAG assembly and modification. For example, there are four known CS 6‐O‐sulphotransferases^7^, ^47^ responsible for making 6‐O‐sulphated CS structures resulting in the observed D0a6 disaccharide. BLMA5 has opposite effects on D0a6 disaccharide in BLMA5 treated LLC and HCT116 cells, which suggest that the two cell lines either expressed different CS 6‐O‐sulphotransferase(s) or the CS 6‐O‐sulphotransferases were behaved differently in the two cell lines.

Figure 3D‐F showed the majority of the GAGs lacked a clear concentration dependence on the cell lines tested, which demanded an explanation. In fact, GAGs are a mixture of molecules with varying molecular weight, charge density and specific sequences. The biological functions of GAGs are charge density‐dependent, sequence‐dependen, or both charge density‐ and sequence‐dependent. The biological effects of GAGs are not always linear with increased GAG concentrations even in a biochemical assay. The bell‐shaped concentration dependence of GAGs is common in cell‐based assays.^41^, ^48^ Among all the GAGs, heparin is the mostly charged and also has the rare 3‐O‐sulphated sequences that are critical for its anticoagulant activities. Heparin is the most active GAG in most of biological tests but with exceptions*.* 49 Indeed, the data in Figure 4 showed that heparin appeared less effective than HS in inhibiting BLMA5‐induced cytotoxicity, indicating HS was involved in the uptake of BLMA5.

With the same genetic background, BLMA5 showed little toxicity even at 160 μmol/L in the cell surface GAG‐deficient CHO 745 cells compared to that of CHO K1 and CHO 3.1 cells. These experimental results indicated that GAGs may participate in the uptake of BLMA5 through a cell surface GAG‐dependent manner. The occurrence of pulmonary fibrosis induced by bleomycin is a major clinical problem.^50^, ^51^, ^52^ Resistance to bleomycin in cancer cell lines is characterized by prolonged doubling time, reduced DNA damage and evasion of G2/M arrest and apoptosis,^52^ but CHO 745 cells have the same doubling time as that of CHO K1 and CHO 3.1 cells.^1^, ^36^, ^53^ Based on these results, we propose that GAGs in lung epithelial cells may help cells to uptake BLMA5. We have established a BLMA5‐induced mouse pulmonary fibrosis model and this speculation is under investigation.

Glycosaminoglycan assembly in the Golgi is sensitive to drug treatment and a variety of environmental factors because there is no template for GAG biosynthesis. However, the drug and environmental induced structural changes of GAGs are an understudied area due to lack of proper methods. Therefore, the flow cytometry‐based cell surface FGF/FGFR/GAG binding assay plus the method of stable isotope labelling coupled with LC‐MS analysis of GAG disaccharide compositions used in current study should be effective tools to search for small molecule therapeutics that alter GAG biosynthesis in future.^4^, ^54^, ^55^

### 4.1 Statistical analysis of data

All statistical calculations were done using IBM SPSS Statistics 21. All data are represented as the mean ± SD *t* test was used to determine the possible significant differences (*P* < .05) of indicators between control group and treatment groups.

## CONFLICT OF INTEREST

The authors declare that they have no competing interests.

## AUTHOR CONTRIBUTIONS

YL and LZ designed the study. YL, XL, YL and YH performed the experiments and analysed the data. CH, HW, JL, GZ, SZ and AZ contributed reagents/materials/analysis/interpretation of the data. YL and LZ wrote the paper.

## Data Availability

The data in the current study are available from the corresponding authors on reasonable request.
